# Comparison of the Effectiveness of Probiotic, Chlorhexidine-based Mouthwashes, and Oil Pulling Therapy on Plaque Accumulation and Gingival Inflammation in 10- to 12-year-old Schoolchildren: A Randomized Controlled Trial

**DOI:** 10.5005/jp-journals-10005-1487

**Published:** 2018-04-01

**Authors:** Saravana K Kandaswamy, Asokan Sharath, PR Geetha Priya

**Affiliations:** 1 Postgraduate Student, Department of Pedodontics and Preventive Dentistry, K.S.R. Institute of Dental Science and Research, Tiruchengode, Tamil Nadu, India; 2 Professor and Head, Department of Pedodontics, K.S.R. Institute of Dental Science and Research, Tiruchengode, Tamil Nadu, India; 3 Reader, Department of Pedodontics, K.S.R. Institute of Dental Science and Research, Tiruchengode, Tamil Nadu, India

**Keywords:** Chlorhexidine, Gingival index, Plaque index, Probiotic, Sesame oil.

## Abstract

**Introduction:**

The use of a mouthwash augments mechanical removal of plaque by brushing and flossing and helps maintain oral health through its antiplaque and antibacterial chemical properties.

**Aim:**

To evaluate the effectiveness of a probiotic mouthwash, sesame oil pulling therapy, and chlorhexidine-based mouth-wash on plaque accumulation and gingival inflammation in schoolchildren aged 10 to 12 years.

**Materials and methods:**

The randomized controlled trial included 45 healthy schoolchildren aged 10 to 12 years and studying in Government High School, Tiruchengode, Tamil Nadu, India. The participants were randomly divided into three groups, I, II, and III, with 15 children in each group as follows: group I: probiotic mouthwash; group II: chlorhexidine mouthwash; and group III: sesame oil. Baseline scores of plaque index (PI) and modified gingival index (GI) were recorded followed by a full mouth oral prophylaxis. The designated mouth rinses were distributed to the respective groups and they were instructed to rinse once daily. Their parents supervised the children during the use of mouthwash. On the 15th and 30th day, the children were subjected to the same clinical measurements. Children’s acceptance of their plaque control method was assessed using a modified facial image scale.

**Results:**

Intragroup comparisons for both the GI and PI scores were statistically significant (p ≤ 0.001) in all the three groups. Difference in the GI scores between the 15th and 30th day was statistically significant for chlorhexidine group alone (p = 0.024). Intergroup comparisons between the three groups were not statistically significant.

**Conclusion:**

Probiotic mouthwash, chlorhexidine mouthwash, and sesame oil were equally effective in reducing plaque and in improving the gingival status of children. The difference between the gingival scores on the 15th and 30th day was statistically significant in the chlorhexidine group.

**How to cite this article:** Kandaswamy SK, Sharath A, Priya PRG. Comparison of the Effectiveness of Probiotic, Chlorhexidine-based Mouthwashes, and Oil Pulling Therapy on Plaque Accumulation and Gingival Inflammation in 10- to 12-year-old Schoolchildren: A Randomized Controlled Trial. Int J Clin Pediatr Dent 2018;11(2):66-70.

## INTRODUCTION

Plaque control measures employ a variety of mouthwashes to augment mechanical removal by inhibiting or reducing plaque accumulation and gingival inflammation. Chlorhexidine gluconate is considered the most effective antiplaque and antigingivitis agent. But Flotra et al have reported side effects like discoloration of teeth and tongue, oral mucosal erosion, taste disturbances, and paresthesia associated with its usage.^1^ Metchnikof propagated the idea of probiotics by proposing a diet containing milk fermented by lactobacilli that could probably increase human life.^2^ Guarner et al stated that administering live organisms in adequate amounts confers a positive health benefit for the host.^2^ Probiotic technology is considered a breakthrough approach in oral health domain, which involves utilizing natural beneficial oral flora as a defense mechanism against detrimental bacteria.^3^ Haukioja^4^ reported that no conclusive evidence is available on the effect of probiotics on dental caries. Though studies report significant improvement in gingival and periodontal conditions, most trials used probiotics for a short period of time of 14 days. Oil pulling is a traditional Indian folk remedy called “Kavala Gandoosha” in Ayurvedic literature, familiarized by Dr Karach in the 1990s in Russia. Hebbar et al^5^ have reported a definite reduction in incidence of dental caries besides prevention of halitosis and xerostomia. Asokan et al^6^ reported significant improvement in plaque and gingival status in young adolescents using oil pulling therapy. This study was planned to compare the effectiveness of a probiotic and a chlorhexidine-based mouth rinse against traditional oil pulling therapy with sesame oil for a period of 30 days.

## MATERIALS AND METHODS

A clinical trial was planned and the protocol was approved by the institutional review board and institutional ethics committee. Informed consent, both written and verbal, was obtained from both the parent and the children and the school authorities. The inclusion criteria were schoolchildren aged 10 to 12 years with no recent antibiotic therapy for the past 4 weeks. Children undergoing orthodontic treatment, those suffering from any systemic illness or using any other mouth rinses, or oral hygiene aids other than routine toothbrushing were excluded.

Forty-five schoolchildren from Government High School, Tiruchengode, Tamil Nadu, India, were included in the study. Baseline plaque score was calculated using the PI (Silness and Loe) and gingival status was determined using the modified GI (Lobene). All the children were subjected to oral prophylaxis and randomly divided into three groups I, II, and III using a computer-generated sequence in the ratio of 1:1:1. Group I was provided with probiotic mouthwash, while group II was provided with chlorhexidine mouthwash and group III with sesame oil. The designated mouthwashes were distributed to the respective groups; 10 mL of chlorhexidine mouthwash (Rexidin, Warren-Indoco Remedies Ltd, Mumbai, India) was diluted in equal amount of water and rinsed for 1 to 2 minutes nearly 30 minutes after tooth brushing. Bifilac sachets (Allianz Biosciences Pvt Ltd, Puducherry, India) containing the probiotic powder were diluted in 10 mL of distilled water and used as a mouthwash. Sesame oil (Idhayam group of companies, Virudhunagar, India) available in 10 mL sachets were given and oil pulling was done for approximately 5 minutes prior to brushing. Children in groups I and II were instructed not to eat, drink, or rinse for 30 minutes after using the mouthwash. Parents were asked to supervise their children during the use of mouthwash. Plaque and gingival scores were again recorded on the 15th and 30th day respectively. Children’s acceptance of their plaque control method was assessed by using a modified facial image scale. It had three smiley faces: (a) happy face showing that they liked the method, (b) neutral face showing that they neither liked it nor disliked it, and (c) sad face showing that they did not like their method.

### Statistics

Statistical Package for the Social Sciences version 17 was used for analysis with statistical significance set at p ≤ 0.05. Intragroup comparison among the three groups was calculated by using Friedman’s test. The difference between the PI and GI scores on the 15th and 30th day and the baseline was deducted and assessed using the Wilcoxon sum-rank test. Kruskal-Wallis analysis of variance test was used for assessing intergroup comparison.

## RESULTS

The mean GI score for the probiotic group was 0.320 ± 0.246 at baseline, which was reduced to 0.040 ± 0.83 at 30 days; for the chlorhexidine group, it reduced from 0.373 ± 0.266 to 0.053 ± 0.092 and for sesame oil it reduced from 0.062 ± 0.375 to 0.080 ± 0.101, as shown in Table 1. Similarly, the mean plaque score for the probiotic group reduced from 0.966 ± 0.319 to 0.360 ± 0.266 in 30 days; for the chlorhexidine group, it reduced from 0.866 ± 0.255 to 0.180 ± 0.193; and for the sesame oil, it reduced from 0.860 ± 0.468 to 0.240 ± 0.252, as shown in Table 2. Difference in the GI scores between the 15th and 30th day was statistically significant for chlorhexidine group alone (p-value = 0.024), as shown in Table 3. Intergroup comparison at all time periods between the three groups was not statistically significant and is shown in Table 4.

**Table 1:** **Table 1:** Intragroup comparison of GI score

*Groups*				*n*		*Mean ± SD*		p-value*	
Probiotic		Baseline—GI		15		0.320 ± 0.246		<0.001	
		15th day—GI		15		0.047 ± 0.099			
		30th day—GI		15		0.040 ± 0.083			
Chlorhexidine		Baseline—GI		15		0.373 ± 0.266		<0.001	
		15th day—GI		15		0.247 ± 0.297			
		30th day—GI		15		0.053 ± 0.092			
Sesame oil		Baseline—GI		15		0.627 ± 0.375		<0.001	
		15th day—GI		15		0.147 ± 0.210			
		30th day—GI		15		0.080 ± 0.101			

*Friedman test; SD: Standard deviation

**Table 2:** **Table 2:** Intragroup comparison of PI score

*Groups*				*n*		*Mean ± SD*		p-value*	
Probiotic		Baseline—PI		15		0.966 ± 0.319		<0.001	
		15th day—PI		15		0.520 ± 0.211			
		30th day—PI		15		0.360 ± 0.266			
Chlorhexidine		Baseline—PI		15		0.886 ± 0.255		<0.001	
		15th day—PI		15		0.553 ± 0.320			
		30th day—PI		15		0.180 ± 0.193			
Sesame oil		Baseline—PI		15		0.860 ± 0.468		<0.001	
		15th day—PI		15		0.620 ± 0.436			
		30th day—PI		15		0.240 ± 0.252			

*Friedman test; SD: Standard deviation

**Table 3:** **Table 3:** Comparison of the difference in GI score, PI score at various time periods within the three groups

		*GI score*						*PI score*					
		*GI (B)-GI (15)*		*GI (B)-GI (30)*		*GI (15)-GI (30)*		*PI (B)-PI (15)*		*PI (B)-PI (30)*		*PI (15)-PI (30)*	
*Groups*		p-value*											
Probiotic		0.003		0.004		0.78		0.001		0.002		0.037	
Chlorhexidine		0.004		0.002		0.024		0.001		0.001		0.001	
Sesame oil		0.003		0.001		0.204		0.016		0.005		0.001	

B: Baseline; 15: 15th day; 30: 30th day; *Wilcoxon sum rank test

**Table 4:** **Table 4:** Intergroup comparison between the three groups for both GI and PI scores

*Intergroup comparison*				*n*		*Mean ± standard deviation*		p-value*	
Baseline—GI		Probiotic		15		0.320 ± 0.246		0.052	
		Chlorhexidine		15		0.373 ± 0.266			
		Sesame oil		15		0.627 ± 0.375			
15th day—GI		Probiotic		15		0.047 ± 0.099		0.115	
		Chlorhexidine		15		0.247 ± 0.297			
		Sesame oil		15		0.147 ± 0.210			
30th day—GI		Probiotic		15		0.040 ± 0.083		0.477	
		Chlorhexidine		15		0.053 ± 0.092			
		Sesame oil		15		0.080 ± 0.101			
Baseline—PI		Probiotic		15		0.967 ± 0.320		0.454	
		Chlorhexidine		15		0.887 ± 0.256			
		Sesame oil		15		0.860 ± 0.469			
15th day—PI		Probiotic		15		0.520 ± 0.211		0.976	
		Chlorhexidine		15		0.553 ± 0.320			
		Sesame oil		15		0.620 ± 0.436			
30th day—PI		Probiotic		15		0.360 ± 0.267		0.117	
		Chlorhexidine		15		0.180 ± 0.193			
		Sesame oil		15		0.240 ± 0.253			

*Kruskal-Wallis analysis of variance

## DISCUSSION

Mechanical means of plaque removal by brushing and flossing is the most effective method to prevent plaque formation and dental caries. The use of a mouthwash augments maintenance of oral health through its antiplaque and antibacterial chemical properties.^7^

Chlorhexidine gluconate is the most widely used mouthwash, as it is effective against various pathogenic microorganisms, fungi, yeasts, and viruses. Its mechanism of action is by alteration of the cell membrane of the bacteria and it owes its antiplaque effect to its substantivity and pin cushion effect.^8^ Mechanism of action of chlorhexidine occurs in three ways. It blocks the acidic groups of salivary glycoprotein, adsorbs to the extracellular polysaccharides of bacteria, and reduces its ability to bind to tooth surfaces, and lastly by competing with calcium ion agglutinating factors in plaque.^9^ But it has some disadvantages like discoloration of teeth and tongue, oral mucosal erosions, and taste disturbances, which resulted in lookout for other combinations of mouthwashes as an alternative.^8^

In recent days, probiotics has drawn the attention of researchers worldwide for its potential benefits in maintenance of oral health. Natural beneficial bacteria are administered to compete and provide a natural defense against the harmful microorganisms. Probiotic strains contain bacterial strains which are not harmful, do not develop antibiotic resistance, and are nontoxic.^10^ In the present study, the probiotic (Bifilac) used contained 30 millions spores of *Streptococcus faecalis* T-110 per 0.5 gm, 2 million spores of *Clostridium butyricum,* 1 million spores of *Bacillus mesentericus* TO-A, and 50,000 spores of *Lactobacillus sporogenes.* Jothika et al^8^ have shown that there was reduction in colony forming units of Streptococcus strains in plaque samples with usage of probiotic mouth-wash. The reduction was similar to chlorhexidine and fluoridated mouthwashes.^9^ Shah^11^ compared the effectiveness of probiotic, chlorhexidine, and fluoride-based mouthwashes in 6- to 10-year-old children and found promising results with usage of probiotic mouthwash in plaque reduction and improvement of gingival status.

Oil pulling therapy involves swishing of oil which has been an ancient ayurvedic practice and is said to confer both oral and general health benefits.^12^ Studies by Amith et al^15^ and Sharath et al have shown the effectiveness of oil pulling therapy against plaque-induced gingivitis. The exact mechanism of action in plaque reduction is not known. It is believed that the viscosity of the oil inhibited bacterial adhesion and plaque coaggregation. One of the suggested mechanisms of action of oil therapy is saponification or “soap-formation” process that occurs due to alkali hydrolysis of fat.^13^ Hebbar et al^5^ stated that absence of a lingering taste, nonstaining of teeth, and no allergic reactions are added advantages for oil pulling besides being simple and easy to use. With encouraging results of probiotic mouthwash from short-term trials and promising beneficial effects conferred by oil pulling therapy, the present study attempted to compare the effectiveness of these two plaque control methods against chlorhexidine gluconate, which is the gold standard mouthwash for decades.

Harini and Anegundi^2^ found significant reduction in the GI score, with the probiotic group (mean GI score = 0.2300) being better than the chlorhexidine group (mean GI score = 0.6805). The authors attributed the antiplaque effect of probiotics to its ability in reducing bacterial adhesion resulting in diminished bacterial growth and proliferation. There was a statistically significant difference in the chlorhexidine group alone, when difference in GI scores was calculated between the 15th and 30th day in the present study. Purunaik et al,^3^ in their 14-day trial, reported a greater reduction in plaque score in the chlorhexidine group compared with the probiotic group. But the GI score for probiotic mouth rinse was more effective than chlorhexidine (p < 0.01), which is contrary to the results of the present study (p-value = 0.024).

Mishra et al^14^ reported that herbal and chlorhexidine mouthwashes were more effective than probiotic mouth rinse in children between 6 and 14 years of age when used for a period of 7 days. The low efficacy of probiotic rinse according to the researchers is probably due to the mutual exchange of microbes in the plaque layer, the virulent bacteria replaced by the probiotic bacteria themselves. But the present study showed that all three treatment modes were equally effective and no statistically significant difference between the three groups was observed.

Amith et al^15^ have reported that oil pulling effectively reduced PI and GI scores in students aged 19 to 21 years of age in a 45-day trial run. Sharath et al showed that oil pulling therapy with sesame oil is equally effective as chlorhexidine (p < 0.05) in adolescent children between 16 and 18 years of age. A greater reduction in colony count of microorganisms in the plaque sample was recorded in oil pulling group.^13^

In this study, a modified facial image scale was used to rate children’s acceptance of their plaque control method. Only three smiley faces indicating happiness, sadness, and neutrality were given to the children to make it easier for them to choose and to eliminate bias. Interestingly, sesame oil had a greater acceptance of 85% compared with the other two groups; 80% acceptance was seen in the chlorhexidine group, while it was only 56% in the probiotic group. Although oil pulling procedure was more time-consuming, children in sesame oil group found greater liking for their plaque removal method. This could be probably attributed to the familiarity of the oil’s taste, since sesame oil is the most commonly used oil for cooking in Tamil Nadu. Children reported dislike toward taste and odor of the probiotic powder, which was probably the reason for poor acceptance among this group of children. Considering cost-effectiveness, sesame oil cost was the cheaper mode costing approximately 4 rupees per day. The cost of chlorhexidine mouthwash was 5 rupees per day, while the probiotic sachet costed 12 rupees per day. Considering both the child’s acceptance and cost-effectiveness, sesame oil can be considered as an effective adjunct and a viable alternative for chlorhexidine.

A longer trial with a cross-over design and adequate washout period is warranted. The cross-over design would help to assess the child’s acceptance and preference after exposure to all the three plaque control measures. Within the limitations of this study, it is possible to conclude that:

- The use of probiotic mouthwash, chlorhexidine mouthwash, and sesame oil pulling therapy were equally effective in reduction of plaque and in the improvement of the gingival status of children.
- Greater acceptance of oil pulling therapy and chlorhexidine mouthwashes was seen compared with the probiotic mouthwash. Sesame oil could be a suitable alternative for chlorhexidine.
- The cost factor may play a big role in the daily usage of the mouthwash and probiotics may not be a viable alternative, especially in lower socioeconomic strata.
